# ﻿Morphological and molecular evidence reject conspecificity of Malagasy and Mascarene *Parablechnum* (Polypodiopsida, Blechnaceae)

**DOI:** 10.3897/phytokeys.214.95125

**Published:** 2022-11-25

**Authors:** Sonia Molino, Irene Lafuente, Germinal Rouhan, Rafael Medina

**Affiliations:** 1 Department of Biodiversity, Ecology and Evolution, Universidad Complutense de Madrid, Calle Jose Antonio Novais 12, 28040 Madrid, Spain Universidad Complutense de Madrid Madrid Spain; 2 Institut de Systématique, Evolution, Biodiversité (ISYEB), Muséum national d’Histoire naturelle, CNRS, Sorbonne Université, EPHE, Université des Antilles, Paris, France Sorbonne Université Paris France

**Keywords:** biogeography, Madagascar, *Parablechnumhumbertii*, *Parablechnummarginatum*, Réunion, sporangiasters

## Abstract

Under the current treatment of the Blechnaceae, only one species of the fern genus *Parablechnum* is recognised in the western Indian Ocean, often referred to as *P.marginatum*. Two varieties are currently recognised within it: a type variety present in the Mascarene Islands of Réunion and Mauritius and P.marginatumvar.humbertii in Madagascar. Recent molecular evidence suggested that these two varieties are not closely related, questioning their conspecific status. To collect further evidence to support a taxonomic decision, we performed a morphological study based on 57 herbarium specimens comparing traits from general morphology, cross section of the fertile pinnae, sporangia and spores. As a result, Malagasy specimens can be distinguished morphologically from the Mascarene ones by pinna apex and pinna section, the presence of sporangiasters and spore ornamentation. Additionally, spore size analyses resulted in statistically significant differences between both varieties. Our results, aligned with the available phylogenetic data, support that these two taxa should be recognised as separate species and, hence, we propose the necessary new combination and provide full descriptions.

## ﻿Introduction

*Parablechnum* C.Presl is the most diverse genus within the fern family Blechnaceae, with about 65 species (Gasper et al. 2016) whose range shows two major centres of diversity, one in Meso and South America and a second one centred in Eastern Australasia. Currently, only two species are considered native outside these centres (Rakotondrainibe et al. 2013; Gasper et al. 2016), one present in southern and south-eastern Africa, *Parablechnumcapense* (Burm.f.) Gasper & Salino and a second one native to the western Indian Ocean (Madagascar and the Mascarenes), *Parablechnummarginatum* (Kuhn) Gasper & Salino. Two varieties are currently considered within the latter (Rakotondrainibe et al. 2013; Gasper et al. 2016). The type variety, Parablechnummarginatumvar.marginatum, is present in Réunion, from where the type was originally collected and was also collected in Mauritius (Appendix 1). A second variety, P.marginatumvar.humbertii (Tardieu) Gasper & Salino accommodates the specimens found in Madagascar, originally considered as a distinct species by Tardieu-Blot (1955). The taxonomic rank of these two varieties, however, needs a reconsideration following recent molecular studies (Bauret 2017), where the specimens from Madagascar and Réunion did not cluster together in a monophyletic group. Here, we reassess the taxonomic status of the western Indian Ocean *Parablechnum* after a morphological examination of herbarium specimens of the two taxa.

## ﻿Materials and methods

We examined 57 herbarium specimens from Herb. P (Appendix 1). We observed the general morphology (i.e. shape of the frond, scales, axes etc.); anatomy of the fertile pinnae; morphology of the sporangia; and size and ornamentation of the spores. Microscopy work followed Gurr (1966) and Ruzin (1999).

For the anatomy of the fertile pinnae, we performed cross-sections in at least two individuals of both varieties. The samples were softened for approximately 5 minutes in water and then manually cross-sectioned in the middle area of the fertile pinnae. The sections were then rinsed by immersion in a 50% solution of sodium hypochlorite for 2–5 minutes. After several washes with water, the sections were stained with 0.1% aqueous toluidine blue (TBO). All microscopic pictures were taken with a Nikon Eclipse Ci microscope with a Nikon DS-Fi2 camera.

Sporangia analysis was carried out by scraping the sori of the previously softened and rinsed pinnae. The protocol and terminology followed Prada et al. (2016), Molino et al. (2020) and Wal et al. (2021). Spores and sporangia were mounted directly in water, imaged under the optical microscope and measured with the *Piximètre* software (Henriot and Cheype 2022). We measured at least 30 spores from three different individuals of the two varieties, excluding the perispore and at least three sporangia from three different individuals of the two taxa. With the spore data, we calculated the shape and volume of each spore following the formulae of Barrington et al. (1986, 2020).

Spore and sporangium measurements were used to perform descriptive statistics and mean comparisons in R using the R Commander package (Fox 2005). Data were tested for normality using the Shapiro-Wilks normality test (Shapiro and Wilk 1965). For those that fit a normal distribution (spore volume), mean comparison was performed with a one-factor ANOVA and for those data that did not fit a normal distribution (spore length, width and shape), we performed a Mann-Whitney U test (Wilcoxon 1945; Mann and Whitney 1947).

From two individuals of each variety, we studied spore ornamentation through scanning electron microscopy (**SEM**). The samples were mounted in a sample holder with carbon adhesive, metallised with gold and observed in a SEM JSM 6400 JEOL operating at 20 kV. The observations were made at the National Center of Electronic Microscopy (**CNME**) of Universidad Complutense de Madrid. Photographs of details at a more macromorphological level, such as fronds or scales, were taken with a Leica Stereozoom S9i with Swing Arm Stand stereomicroscope.

## ﻿Results

**Figure 1.**
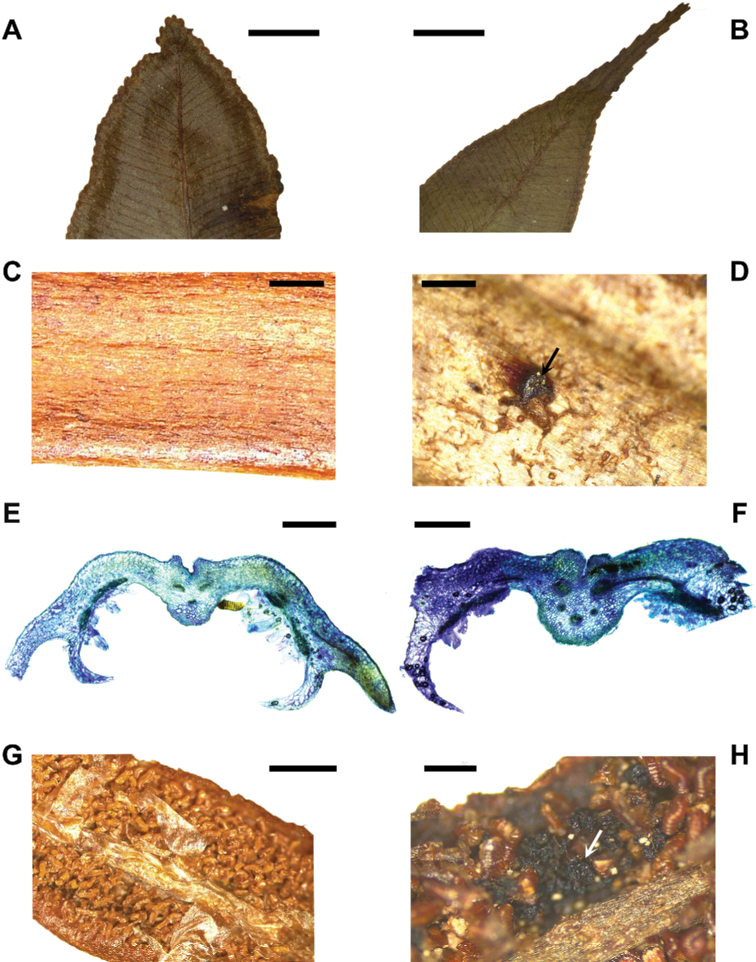
Details of the traits observed in *P.marginatum* var. *Marginatum* (A, C, E, G) and P.marginatumvar.humbertii (B, D, F, H) **A** apex of a sterile pinna in the var. *marginatum*, adaxial surface (*Cowemoy s.n.*, P01462834) **B** apex of a sterile pinna in var. humbertii, adaxial surface (*Rakotondrainibe 1673*, P00100192) **C** petiole surface in the var. *marginatum* (*Cadet 4050B2*, P01462767) **D** petiole surface in var. humbertii with a scar left by a scale pointed with an arrow (*Rakotondrainibe 1673*, P00100193) **E** fertile pinna cross section of the var. *marginatum* (*Bradé 958*, P00917035) **F** fertile pinna cross section of var.humbertii (*Rakotondrainibe & Raharimalala 2519*, P00904704) **G** sorus in the var. *marginatum* (*Cadet 4050B1*, P01462768) **H** sori of var. humbertii, with sporangiasters pointed with an arrow (*Rakotondrainibe 2743*, P00059959). Scale bar: 5 mm (**A**); 2.5 mm (**B**); 1 mm (**C, D**); 800 µm (**E, F**); 2 mm (**G**); 500 µm (**H**).

Our morphological analysis shows qualitative and quantitative differences between the two taxa that are summarised in Table 1 and the full descriptions given in the Nomenclature section. There are some differences in the sizes of the two taxa and in some characters, such as the sometimes slightly creeping rhizomes in the case of the P.marginatumvar.marginatum (vs. erect or suberect in var. humbertii). However, we believe that the most reliable characters that easily distinguish the two taxa are the obtuse or acute apices on both sterile and fertile pinnae of P.marginatumvar.marginatum (Fig. 1A) vs. the long-acuminate apices in both sterile and fertile pinnae of var. humbertii (Fig. 1B) and the smooth petiole surface in P.marginatumvar.marginatum (Fig. 1C) vs. petioles with scars left by the scales in var. humbertii (Fig. 1D).

**Table 1.** Summary of the most useful traits to distinguish Parablechnummarginatumvar.marginatum from P.marginatumvar.humbertii.

Taxon	Pinnae apices (Fig. 1A, B)	Petiole surface (Fig. 1C, D)	Number of bundles in the costa (Fig. 1G, H)	Sporangiasters (Fig. 1E, F)	Spore ornamentation (Fig. 2)
P.marginatumvar.marginatum	Caudate	Smooth	5	Absent	Perisporium forming defined areolae, with filaments forming a net
P.marginatumvar.humbertii	Long attenuate	With scars left by the scales	3	Present	Perisporium not forming defined areolae but a maze, filaments occasional

Fertile pinnae of P.marginatumvar.marginatum present a costa, grooved adaxially and prominent abaxially, with three vascular bundles, elongated receptacle in the sori, covered by a short, complex indusium (composed by more than one cell layers), which arises at approximately one third of the distance between the margin and the costa, leaving a sterile portion towards the margin. The margin of the pinna is thick (Fig. 1E). Variety *humbertii* presents a costa grooved adaxially and prominent abaxially, with five vascular bundles and elongated receptacle in the sori, covered by long complex indusium, which arises on the first third of the distance between the margin and the costa, leaving a very small sterile portion of the margin (Fig. 1F). We consider that the different number of vascular bundles in the cross section of the fertile pinnae is the most distinctive diagnostic character.

Both taxa present monolete spores, with an ellipsoid outline in the polar view and flat-convex to concave-convex (reniform) in the equatorial longitudinal view. The spores of each taxon are described below. Sizes are rounded values; the exact values with their standard deviation can be found in Table 2, together with shape (length/width ratio) and estimated volume.

**Table 2.** Characterisation of the spores of both taxa. The mean ± standard deviation is presented.

Taxon	Spore length (μm)	Spore width (μm)	Shape	Volume (μm^2^)
P.marginatumvar.marginatum	64.32 ± 7.22	45.67 ± 6.83	1.42 ± 0.16	7.3435.74 ± 2.6210.03
P.marginatumvar.humbertii	66.14 ± 5.42	48.74 ± 5.62	1.37 ± 0.12	8.4367.01 ± 2.3689.94

**Figure 2.**
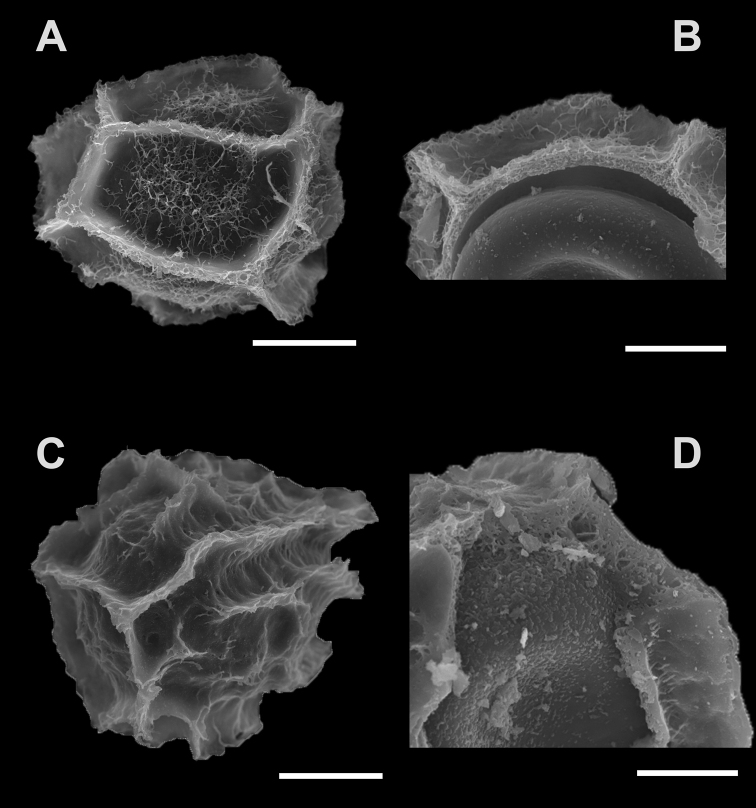
Spores of Parablechnummarginatumvar.marginatumunder SEM**A** spore (*Cowemoy s.n.*, P01462832) **B** detail of the internal structure of the perispore and the exospore (*Lorence s.n.*, MO2715099). Spore of P.marginatumvar.humbertii under SEM**C** spore (*Rakotondrainibe 3571*, P0085125) **D** detail of the internal structure of the perispore and the exospore (*Rakotondrainibe 3571*, P00085125). Scale bar: 25 µm (**A, C**); 14 µm (**B**); 12 µm (**D**).

Spores of P.marginatumvar.marginatum: (41‒) 64 (‒81) × (27‒) 46 (‒60) µm, perisporium folded cristate-reticulate, with protruding ridges and with large areas between them (areolae), measuring approximately 30 µm, covered with filamentous micro-ornamentation forming a kind of net that is arranged on a nearly smooth surface; internal structure of the perisporium of spongy appearance and irregularly granular exosporium (Fig. 2A, B).

Spores of P.marginatumvar.humbertii: (49‒) 66 (‒78) × (35‒) 49 (‒62) µm, perispore folded cristate-reticulate, with protruding ridges, but without large and regular areas between them, but rather irregular corridors, without filaments or with moderately abundant filaments distributed over the entire surface; internal structure of perispore spongy in appearance and exosporium regularly granular (Fig. 2C, D).

After comparison of means using the tests specified above, we obtained significant differences for all characters between the two taxa (spore length W = 6268.5, p-value = 0.047; spore width W = 5588.5, p-value = 0.0014; shape W = 8.959, p-value = 0.0057; volume F = 10.56, p-value = 0.001), suggesting that the spores of P.marginatumvar.humbertii are significantly larger than those of the var. marginatum. However, we believe that the best spore character to differentiate these taxa is the perispore ornamentation, as, although there are significant differences in spore sizes, the ranges overlap (Fig. 2, Table 2). Spore ornamentation in P.marginatumvar.marginatum form defined areolae and present a net of filamentous processes, while in var. humbertii, they do not form areolae, but corridors and filamentous processes are occasional.

The sporangia of both taxa are leptosporangiate, with pedicels of 2–3 rows of cells with a rosette joining them to a nearly spherical capsule with a vertical arc interrupted by a stomium. No posterior basal cells were observed. Table 3 summarises the morphometric variation of the sporangia. Additionally, the sporangia of P.marginatumvar.humbertii are intermixed with shorter, darker, sterile sporangia that we interpret as sporangiasters (Fig. 1H). None of the sporangium characters showed significant differences between the two taxa (number of arc cells F = 3.237, p-value = 0.084; arc width F = 2.307, p-value = 0.142; capsule length W = 93, p-value = 0.905; capsule width F = 3.457, p-value = 0.075; number of lip cells W = 45, p-value = 0.117; upper lip width F = 0.087, p-value = 0.771; lower lip width F = 1.493, p-value = 0.237; number of epistomium cells W = 92, p-value = 0.3; number of hypostomium cells F = 0, p-value = 0.983; pedicel length F = 1.019, p-value = 0.319; rosette length F = 0.567, p-value = 0.455).

**Table 3.** Characterisation of the sporangia of both taxa. The mean ± standard deviation is presented, all the values are in μm. Arc = number of cells in the arch; Arc wd = thickness of the arch; Cap = size of the sporangia capsule (length x width); Lip = number of cells forming the lip (stomium); Sup = upper lip cells width; Inf = lower lip cells width; Epi = number of cells in the epistomium; Hyp = number of cells in the hypostomium; Ros = rosette length; Ped = pedicel length.

Taxon	Arc	Arc wd	Cap	Lip	Sup	Inf	Epi	Hyp	Ros	Ped
P.marginatumvar.marginatum	22.9 ± 2.4	79.8 ± 9.9	441 ± 71.3 × 258.5 ± 26.6	4 ± 1.1	52. 4 ± 16. 5	54.6 ± 17.3	3.5 ± 1.1	2.5 ± 0.8	59. 3 ± 17.7	561.7 ± 110.7
P.marginatumvar.humbertii	21.4 ± 2	74.4 ± 7.7	414.8 ± 73.4 × 278.1 ± 27.9	4.7 ± 1.1	54.5 ± 17.3	46 ± 12.3	3.1 ± 0.9	2.5 ± 0.8	55.4 ± 18.8	514. 3 ± 189

## ﻿Discussion

To resolve the conspecificity hypothesis of Parablechnummarginatumvar.marginatum and var. humbertii, we have performed a morphological analysis using traits usually showing systematic value within the family Blechnaceae. Regarding the anatomy of fertile pinnae, the study by Prada et al. (2016) defined the characters observable in pinnae cross-sections and showed how these have a high taxonomic value at the generic and specific level, which has been demonstrated in subsequent publications (Molino et al. 2019a, b; Bauret et al. 2020). Spores are a widely used character in fern taxonomy (Tryon and Tryon 1982; Barrington et al. 1986, 2020; Tryon and Lugardon 1990). Spore size, perisporium ornamentation and internal structure are known to be reasonably constant within species, but with considerable variation between species (Lugardon 1974; Tryon and Lugardon 1990). There are numerous studies on the spores of the family Blechnaceae and some of these have been used to successfully discriminate genera and species (Passarelli 2007; Passarelli et al. 2010; Moran et al. 2018; Silva et al. 2019, 2021; Molino et al. 2020; Wal et al. 2021). The genus *Parablechnum* is particularly complicated and the study of spores could be an important element in the delimitation of infrageneric taxa, as has been shown in other studies (Wal et al. 2021). The spores observed for these two taxa present typical ornamentation of the genus: folded cristate-reticulate perispore with or without filamentous processes (Moran et al. 2018). In this case, the ornamentation of the spores serves to distinguish the two taxa.

Sporangia are structures whose ontogeny and variation in characters have been studied for many groups of leptosporangiate ferns (e.g. Bower 1925; Copeland 1947; Wilson 1959). In particular, there have been specific studies in Blechnaceae where sporangia have been key in the separation of genera and species (Prada et al. 2016; Molino et al. 2020; Wal et al. 2021). Although we did not find differences in sporangial characters, their characterisation is novel and may be useful in future studies of the genus *Parablechnum* on a larger scale.

The presence of sporangiasters as a trait with taxonomic value in Blechnaceae was recently observed for the first time in *Parablechnumnesophilum* (T.C.Chambers & P.A.Farrant) Gasper & Salino, a species from Papua New Guinea (Molino et al. 2021). Their occurrence in P.marginatumvar.humbertii seems to also be a useful character to distinguish this taxon from the var. marginatum, suggesting that sporangiasters may be more widespread within the genus than previously thought.

In line with the phylogenetic tree topology obtained by Bauret (2017), this morphological comparison suggests that, as stated for the first time by Tardieu-Blot (1955), Madagascar specimens are not conspecific with those of the Mascarene Islands. In said analysis, P.marginatumvar.humbertii is represented by two accessions and P.marginatumvar.marginatum by another one. The latter is resolved in a clade with 16 American species that is sister to the former. Three maximally supported internal nodes (posterior probability ≥ 0.95, bootstrap ≥ 95) segregate the Mascarene and Malagasy taxa.

Ferns in Madagascar and the archipelagos of the western Indian Ocean may be closely related to lineages from different biogeographic regions (Bauret et al. 2017a, b, 2018; Hennequin et al. 2017; Rouhan and Gaudeul 2021). To date, phylogenetic affinities suggest that *Parablechnum* species of the western Indian Ocean Islands are nested within a clade with many Neotropical taxa, in contrast with *P.capense*, the single continental African species, nested in an Austro-Pacific clade (Gasper et al. 2016, 2017).

Given the relatively recent age of the Blechnaceae (Testo and Sundue 2016) compared to the isolation of Madagascar and the origin of the Mascarenes, long-distance dispersal is the most likely hypothesis for explaining the presence of these taxa in these Islands (Bauret 2017; Bauret et al. 2017a, b, 2018; Rouhan and Gaudeul 2021). Given the topology of the phylogenetic tree (Bauret 2017), a single dispersal event cannot explain the occurrence of the two species in the Malagasy region and, so far, the most likely hypothesis includes two independent events. A richer sampling in an expanded phylogenetic analysis will be critical to resolving the number, origin and timing of the dispersion events to the region.

From the systematic point of view, available information rejects the conspecificity of the two taxa and, hence, we propose that the Malagasy taxon should recover the species rank within *Parablechnum*.

## ﻿Nomenclature and full descriptions

### ﻿ Parablechnum marginatum

Taxon classificationPlantaePolypodialesBlechnaceae

(Kuhn) Gasper & Salino Phytotaxa 275(3): 191–227, 2016.

FFC1012D-EB26-5556-A50E-787816358A41

≡ Blechnummarginatum Kuhn, Filic. Afr.: 92, 1868; Blechnummontbrisonis C.Chr. Index Filic. 157, 1905, nom. nov. for Lomariamarginata Fée, Mém. Foug., 5. Gen. Filic.: 71, 1852, nom. illeg. hom., non L.marginata Schrad., Gött. Gel. Anz. 871. 1824 [≡ Lomariopsismarginata (Schrad.) Kuhn].

#### Type.

Habitat in insulâ Borboniâ, no date, de Montbrison s.n. (not found).

#### Description.

***Plants*** terrestrial; ***rhizomes*** erect, sub-erect or slightly creeping, non-stoloniferous, with ovate to lanceolate scales with elongated apex, more or less filiform, concolorous, brownish, membranaceous, up to 2 cm long; ***fronds*** dimorphic; ***sterile fronds*** with ***petioles*** light brown, grooved adaxially, smooth, up to 50 cm long, with scales in basal zone decreasing in density distally, similar to those of the rhizome, ***laminae*** 1-pinnate, elliptic-acuminate, up to 1 m long, sometimes longer, ***rachises*** light brown, smooth, adaxially grooved, scales similar to those of petiole, more abundant on adaxial side, ***pinnae*** up to 30 pairs, alternate or subopposite, slightly smaller at base, lanceolate to oblong, stalked, becoming basiscopically adnate towards apex of frond, ca. 11 × 2 cm, base asymmetric, subcordate to truncate, apex acute or obtuse, margins slightly serrate, with conforming terminal pinna similar to lateral ones, ***costae*** light brown, grooved adaxially, prominent abaxially, with scales at base similar to those of rachis; ***veinlet*** simple or 1-furcate, patent, catadromous; ***fertile fronds*** larger than sterile ones and more erect, ***petioles*** similar to sterile fronds, ***laminae*** usually up to 50 cm long, lanceolate to oblong, ***rachises*** similar to sterile fronds, ***pinnae*** usually in more pairs than in sterile ones, linear, narrow, ca. 3.0 × 0.2 cm, slightly broader-based, asymmetrical, cordate, apex acute; ***aerophores*** present in both sterile and fertile fronds, tuberculiform, atropurpureus; ***hydathodes*** present in both sterile and fertile fronds, rounded or ovate; ***sori*** linear, continuous, on both sides of costa forming coenosori; ***indusia*** linear, continuous, opening towards costa, dark brown, membranaceous, usually lacerate.

#### Taxonomical notes.

Christensen (1905) proposed *Blechnummontbrisonis* as a replacement name, as the name *Blechnummarginatum* proposed by Kuhn (1868) was based on an illegitimate basionym, *Lomariamarginata* Feé. This name was proposed by Feé (1852) after this combination had already been used by Scharder (1824) for what is now known as *Lomariopsismarginata* (Schard.) Kuhn. However, according to the rules of the current Code (see Art. 6.14 Ex. 18; Turland et al. 2018), *Blechnummarginatum* would be a validly published replacement name for *Lomariamarginata* Feé and, therefore, *Blechnummontbrisonis* would be a superfluous name.

### ﻿ Parablechnum humbertii

Taxon classificationPlantaePolypodialesBlechnaceae

(Tardieu) S.Molino & Lafuente comb. nov.

2233D282-FA13-5AA6-991D-B0D66A5BB9E9

urn:lsid:ipni.org:names:77308765-1

≡ Blechnumhumbertii Tardieu Mém. Inst. Sci. Madagascar, Sér. B, Biol. Vég. 6: 232, f.5, 1955; Blechnummontbrisonis C. Chr. var. humbertii (Tardieu) Rakontondr. Adansonia, série 3, 35(2): 178, 2013. Parablechnummarginatumvar.humbertii (Tardieu) Gasper & Salino Phytotaxa 275(3): 216, 2016.

#### Type.

Madagascar. ‘Vallée de la Lokoho, mont Beondroka, au Nord de Maroambihy, sylve à Lichens, sur gneiss et quartzite’, no date, Humbert 23554 (Holotype: P00483200).

#### Description.

***Plants*** terrestrial; ***rhizomes*** erect or sub-erect, non-stoloniferous, with ovate-lanceolate scales with elongated apex, more or less filiform, concolorous, brownish, membranaceous, with entire margins, up to 20 mm long; ***fronds*** dimorphic, sterile fronds with ***petioles*** 20–30 cm long, dark brown at base, straw-greyish distally, smooth, grooved adaxially, with scales in basal zone decreasing in density distally, similar to those of rhizome, leaving a black scar after falling off; ***laminae*** 1-pinnate, elliptic-acuminate, up to 30 cm, sometimes longer, ***rachises*** light brown, smooth, grooved adaxially, scales similar to those of petiole but narrower, more abundant in the adaxial side, ***pinnae*** in up to 20 pairs, alternate or subopposite, slightly smaller at the base, lanceolate-oblong, stalked, becoming basiscopically adnate towards apex of the frond, 10 × 1.5 cm, base asymmetric, subcordate to truncated, apex long acuminate, margins serrate, with a conforming terminal pinnae similar to lateral ones; ***veinlet*** free, simple or 1-furcate, catadromous; ***fertile fronds*** longer than sterile and more erect, ***petioles*** similar to sterile fronds, ***laminae*** 30 cm long, sometimes longer, lanceolate-oblong, ***rachises*** similar to sterile fronds, ***pinnae*** usually in more pairs than in the sterile ones, linear, narrow, 12.0 × 0.2 cm, slightly broader-based, asymmetrical, cordate, apex long acuminate; ***aerophores*** present in both sterile and fertile fronds, tuberculiform, atropurpureus; ***hydathodes*** present in both sterile and fertile fronds, rounded or ovate; ***sori*** linear, continuous, forming coenosori on both sides of the costa; ***indusia*** linear, continuous, open towards the costa, dark brown, membranaceous, sometimes lacerate.

## Supplementary Material

XML Treatment for Parablechnum marginatum

XML Treatment for Parablechnum humbertii

## ﻿Appendix 1

**Material examined**

***Parablechnummarginatum* (Kuhn) Gasper & Salino**

**Mauritius.** no date, Belanger 98C, (P01462824); no date, Bonpland s.n. (P01532252); ibidem (P01532253); 3 Dec 1909, Meller 6. **Réunion.** no date, no collector (P01462821); ibidem (P01462829); ibidem (P01462833); ibidem (P01557677); ibidem (P01557678); 1892, Cowemoy s.n. (P01462832); ibidem (P01462834); 1875, de Isle 606 (P01462825; P01462826; P01462827); 1842, Lépervanche-Mézière s.n. (P01462830); 1898, Lépervanche-Mézière 17 (P01462771); no date, Lépervanche-Mézière s.n. (P01462820; P01462823; P01462828); 8 Mar 1979, Lorence s.n. (MO3156135; MO3156136); Berge de la Riviére des Marsouins prés Coserne des Hirondelles Nébom, 22 Jul 1973, Cadet 4050B1 (P01462768); ibidem, Cadet 4050B2 (P01462767); Bourbon, no date, Richard 99 (P01462822); cirque de Salazie, sentier vers La Nouvelle, 29 Nov 1973, Badré 1052 bis (P01462769); ibidem, Badré 1055 (P01462836; P01462837); Fourré à Philippia, sentier de la Mare à Joseph au coteau Kerveguen, cirque de Cilaos, 16 Nov 1973, Badré 935 (P01462835, P00917036, P00917037); ibidem, Badré 958 (P00917032; P00917033; P00917034; P00917035); Nationale 3, Bord de route, entre le Col de Bellevue et la Plaine des Palmistes, 5 Nov 2004, Rakotondrainibe & Grangaud 6910 (P00411889); Sentier de Bélouve à la caverne Mussard, no date, Bosser 12208 (P01625974).

***Parablechnumhumbertii* (Tardieu) S. Molino & Lafuente**

**Madagascar. Atsimo-Andrefana**: Eboulis sableux Ambondrombe, 11 Apr 1941, Boiteau 4635 (P02284987; P01632222); **Diana**: Antsiranana, 24 Sep 2015, Bauret et al. 102 (P02435082); Antsiranana, Andapa, Parc National de Marojejy. Aux alentours du Camp 4, au bord de la rivière Andranomifototra, 25 Oct 2011, Rouhan et al. 1209 (P02432741; P02432742; P02432743; P02432744); Antsiranana, Andapa, Befingotra, RS d’Anjanaharibe-Sud, sur le versant Sud-Est, à 12.2 km à l’Ouest-Sud-Ouest de Befingra, 25 Nov 1994, Rakotondrainibe & Raharimalala 2519 (P00904704; P00046988); ibidem, 27 Nov 1994, Rakotondrainibe & Raharimalala 2534 (P00046998); Antsiranana, Andapa, RNI 12 du Marojejy. A 11 km au Nord-Ouest de Manantenina, 27 Oct 1996, Rakotondrainibe 3571(P00085122; P00085123; P00085124; P00085125); Antsiranana, Ambanja, Massif du Manongarivo, Mt d’Antsatrotro, berges de la rivière Ankaramihely, 19 May 1992, Rakotondrainibe 1673 (P00100192; P00100193; P00100194); Antsiranana, Iharana (Vohémar), Marojejy, route vers campement 3, 13 May 2015, Rabarijaona et al. ROM1053 (P00783078; P00783098); **Haute Matsiatra**: Fianarantsoa, Ambalavao, Ambatomboay, RNI d’Andringitra versant E; à environ 38 km au Sud d’Ambalavao, près de la source de la rivière Sahavatoy, 30 May 1995, Rakotondrainibe 2743 (P00059958; P00059959; P00059960; P00059961); **Sava**: Anjanaharibe, 19 Dec 1950, Cours 3772 (P01625801); Vallée de la Lokoho (nord-est), Mont Beondroka au Nord de Maroambihy, 17–22 March 1949 H. Humbert 23554, (P00483200, holotype).
